# Prenatal Predictors of Neurobehavioral Outcome in Children with Fetal Growth Restriction at 6 Years of Age: A Retrospective Cohort Study

**DOI:** 10.3390/children10060997

**Published:** 2023-06-01

**Authors:** Mª José Benítez Marín, Marta Blasco Alonso, Ernesto González Mesa

**Affiliations:** 1 Obstetrics and Gynecology Service, Virgen de la Victoria University Hospital, 29010 Málaga, Spain; 2 Surgical Specialties, Biochemistry and Immunology Department, Málaga University, 29071 Málaga, Spain; martablascoalonso@gmail.com (M.B.A.); egonzalezmesa@uma.es (E.G.M.); 3 Research Group in Maternal-Fetal Medicine, Epigenetics, Women’s Diseases and Reproductive Health, Instituto de Investigación Biomédica de Málaga y Plataforma en Nanomedicina-IBIMA Plataforma Bionand, 29071 Málaga, Spain; 4 Obstetrics and Gynecology Service, Regional University Hospital of Malaga, 29011 Málaga, Spain

**Keywords:** fetal growth restriction, neurodevelopment, behavioral, outcomes, cognitive, brain sparing effect

## Abstract

(1) Background: Fetal growth restriction (FGR) increases the risk of adverse neurodevelopmental outcomes, especially in preterm newborns. This study aims to describe the behavioral results of FGR at 6 years of age and to demonstrate the relationship of certain predictive factors with this development. (2) Methods: This retrospective cohort study included 70 children born in 2015 at the University Hospital Carlos Haya, Málaga, Spain who had been exposed to FGR during pregnancy; neonatal and infant data were recorded retrospectively. Children were assessed prospectively at 6 years of age by means of a strengths and difficulties questionnaire (SDQ) to study behavioral outcomes. (3) Results: We demonstrated that there are higher behavioral disability rates in children exposed to FGR during pregnancy and, in particular, high rates of hyperactivity or conduct problems. We also proved a negative relationship between the birth weight percentile and the total behavioral scale score, along with a positive correlation between hyperactivity and the emotional and behavioral scales. Learning difficulties were more frequent in early-onset FGR than in late-onset FGR. (4) Conclusions: Our study of behavioral development has demonstrated higher behavioral disability rates in children with FGR at 6 years of age; specifically, high rates of hyperactivity or conduct problems. At the same time, we have proved a negative relationship between the birth weight percentile and the total behavioral scale score.

## 1. Introduction

Fetal growth restriction (FGR) has varied greatly in terms of its management over time. Nowadays, FGR management techniques have improved the standard treatment guidelines. As a result, restriction severity and prematurity issues now have established follow-up and completion dates to reduce perinatal morbidity and mortality [1,2,3]. Thanks to improvements in pregnancy management, perinatal care, and neonatal techniques, perinatal mortality has decreased considerably, especially in extremely premature infants. However, these improvements may not appreciably reduce perinatal morbidity [4].

Neonatal outcomes in FGR have been exhaustively researched, and prematurity has been strongly associated with short-term outcomes [2]. Childhood development may also be influenced by FGR [5] and interest in long-term outcomes, specifically regarding neurodevelopment, is growing. A considerable number of studies in human and animal models have demonstrated changes to the nervous system’s structure, affecting brain volume [6,7,8], grey matter volume and structure [8,9,10], and white matter structure and myelinization [11,12], as well as influencing the gyrification process [13] in FGR conditions. These changes may affect motor, cognitive, adaptive, and behavioral development.

However, the results of these studies are often heterogeneous and contradictory. In most cases, assessments may be carried out at very early stages before the development of a given research object’s cognitive deficits or behavioral changes. The studies also include a heterogeneous group of children, both those who are small for their gestational age and those with FGR; in many cases, the authors did not take into account the issue of prematurity, which could alter the results. Despite these heterogeneities and contradictions, outcomes such as poor results in intelligence coefficients, poor academic results, cognitive and emotional alterations, and attention and hyperactivity disorders, as well as behavioral disabilities have been described with early-onset FGR. The most severe cases have been linked with motor disorders and cerebral palsy [6,14,15,16,17,18,19,20]. These results do not shed much light on late-onset FGR when reaching the gestational term [21,22,23,24,25,26,27]. 

Similarly, various research groups have attempted to show the relationship between different prenatal markers and motor, cognitive, and behavioral development. In our previous systematic review, we were able to associate brain sparing with poorer cognitive development. However, the link between brain sparing effect and behavioral skills development was difficult to establish [28].

In our previous research, we assessed FGR children by means of the Battelle Developmental Inventory (BDI), evaluating milestones in different areas. We found a high rate of poor global development, with motor and communication skills being the main areas affected. Conversely, we were able to associate the brain-sparing effect with worse coefficients of global development. However, the cognitive delay rate was low [29].

It has been shown that early attention and early stimulation in children that are born preterm can have a positive effect on motor neurodevelopment, and that this positive effect on cognition continues into school age [30]. Therefore, the early detection of FGR in neurocognitive risk could allow for the implementation of early stimulation strategies to improve their deficits for a longer period. In the same way, cognitive development and emotional intelligence could be improved, and better resilience and future personal relationships obtained, by identifying and improving the influencing factors (both positive and negative).

We believe that children who have been exposed to FGR during pregnancy have a higher risk of behavioral disorders. This study aims to describe the behavioral results of FGR in children at 6 years of age. Secondly, we demonstrate the relationship of certain predictive factors with this developmental issue, which might help us to select a population that is at risk of FGR, in order to assist with early childhood support or with psychological assessment and management.

## 2. Methods

### 2.1. Population

This cohort study had a retrospective design, in which we selected a group of FGR children born in 2015 at University Hospital Carlos Haya, Málaga, Spain. This hospital is a specialized center for the diagnosis and treatment of this pathology. Table 1 describes our inclusion and exclusion criteria, based on the definition of FGR. We define the brain-sparing effect as a cerebroplacental ratio (CPR) below the 5th percentile. When FGR is diagnosed at or below 32 weeks of gestational age, it is defined as early-onset FGR. In the event that this diagnosis is beyond 32 weeks, it is defined as late-onset FGR. Recruitment started in 2021, following approval from the regional ethics committee, and parental consent was obtained before child assessment began. Medical and sociodemographic data were collected from clinical records and parents’ reports. This study protocol was previously described in our most recent publication [29].

### 2.2. Parents’ Reports and Data Collection

We examined the pregnancy and neonatal care information given in the medical records and recorded all the variables related to pregnancy care, FGR characteristics, Doppler measurements, and delivery episodes. After birth, we recorded the variables related to neonatal anthropometric measurements, adverse neonatal outcomes, and, if applicable, admission days in the neonatal intensive care unit (NICU).

Parents completed a survey to provide information on sociodemographic items and childhood problems, such as academic difficulties, the need for early child support, kindergarten attendance, or any major health problems.

### 2.3. Behavioral Assessment

The Spanish version of the strengths and difficulties questionnaire (SDQ) was completed prospectively when the subjects were 6 years of age [31]. This questionnaire provides a brief emotional and behavioral screening test for children that is completed by their parents. It can be used in both low- and high-risk populations [32]. This test consists of 25 items, divided into 5 scales. The first four scales assess negative symptoms (emotional symptoms, conduct problems, hyperactivity and inattention symptoms, and peer relationship problems), while the last scale evaluates positive social relationships (prosocial behavior) [33]. Depending on the scores obtained, children are classified as normal, borderline, or abnormal. For those classified as borderline or abnormal, more in-depth studies are required to diagnose a behavioral problem. We selected this test because it is effective in differentiating between psychiatric and non-psychiatric populations, with the advantage of being shorter than other behavioral tests [34].

At the same time, we assessed the children using a BDI screening test. These results are shown in an earlier article published by our research group [29].

### 2.4. Statistical Analysis

A descriptive analysis was carried out to detail the frequency distribution of the different variables in the cohort, along with the distribution of the behavioral classifications in each scale. We examined the relationship between the sociodemographic variables, Doppler measurements and FGR characteristics, and the behavioral problems in each scale using the chi-squared test. 

Finally, we conducted a multiple linear regression analysis to investigate the effect of (1) sociodemographic variables, (2) FGR characteristics and age at delivery, and (3) neonatal and child outcomes on the score from the total difficulties scale. Normality was tested using a Kolmogorov–Smirnov analysis. Pearson’s coefficient was used for the correlations when we confirmed the normal distribution. For all analyses, we considered a *p*-value below 0.05 to be significant. All data were processed and analyzed with the support of the Statistical Package for the Social Sciences (SPSS), version 22.0 (SPSS Inc., Chicago, IL, USA).

## 3. Results

### 3.1. Population and Characteristics of Population Participants

**Figure 1:**
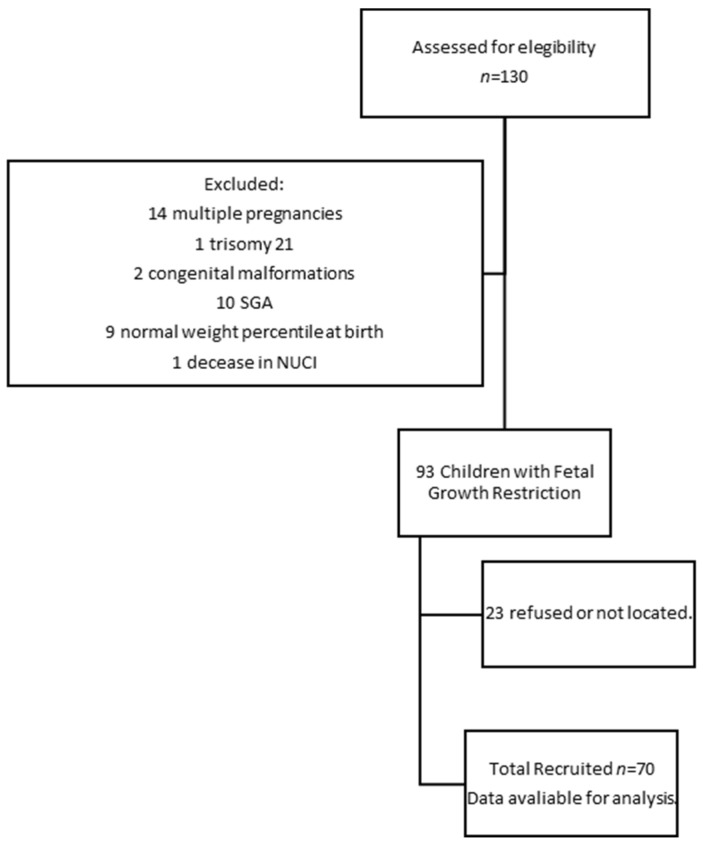
Flow diagram detailing the participants in the research.

One hundred and thirty children diagnosed with FGR during pregnancy who were born in 2015 were initially located. Figure 1 describes our flow diagram of the participants in the research. The study was carried out in a single medical facility; therefore, no sampling of cases was carried out. Consequently, all diagnosed cases of FGR that met the inclusion criteria were considered for the study. Seventy 6-year-old children were recruited, representing the initial population with a confidence level of 95%, a type-II error of 0.6, and a statistical power of 94% [29].

The characteristics of the population participants were described in our previous paper [29]. Table 2 summarizes the sociodemographics, delivery characteristics, and adverse neonatal outcomes of the population.

### 3.2. Behavioral Outcomes at 6 Years of Age

The children were aged 70–84 months at the time of the behavioral test. The average age of the children at the time of assessment was 76.20 months old (SD = 3.70). The learning disabilities rate was 27.1%. Additionally, 36.8% of the children needed early child support, and 16.7% of the children are currently following this program. Parents reported two cases of children who were diagnosed as having an autistic spectrum disorder. One of these children exhibited difficulties in performing adaptive skills, while the other child exhibited a global development disorder. These data were published in our previous paper [29].

Table 3 summarizes the percentages of children with behavioral problems in different areas. We were able to record 30% of the global behavioral disabilities. In the case of hyperactivity symptoms, 30% of the children had an abnormal classification, and 8.6% had a borderline classification. Hyperactivity symptoms and conduct problems were the most common issues.

We found a positive correlation between the total score and the scores obtained on different scales: emotional scale (r = 0.655, *p* ≤ 0.001), behavior scale (r = 0.655, *p* ≤ 0.001), hyperactivity (r = 0.843, *p* ≤ 0.001), and peer problems (r = 0.465, *p* ≤ 0.001). Similarly, a positive correlation was found between the hyperactivity and emotional scale (r = 0.387, *p* = 0.001) and the behavior scale (r = 0.549, *p* ≤ 0.001). Therefore, hyperactive children tended to present more emotional and behavioral problems.

### 3.3. Bivariant Analyses

Table 4 and Table 5 summarize the percentages of children with behavioral problems according to the diagnosis variables. We found no differences in the classification according to the onset time of FGR. However, with the exception of the peer problems scale, there was a tendency of poor results in each scale for the early-onset FGR diagnosis group. Learning difficulties were more frequent in the early-onset FGR group (43.5%) than in the late-onset FGR group (19.6%), reaching statistical significance (χ^2^(1, N = 69) = 4.39, *p* = 0.036). 

Regarding the Doppler measurement variables, we found that a pathological CPR measurement was related to poor results on the full scale. When pathological UA, MCA, and CPR measurements were detected, there was a tendency towards poor classifications in the remaining behavioral scales (no significance).

Gestational age at delivery or birth characteristics were not related to behavioral outcomes. A positive correlation was found between the birth-weight percentile at delivery and the different scales: total score (r = −0.310, *p* = 0.009), emotional scale (r = −0.280, *p* = 0.019), and hyperactivity scale (r = −0.246, *p* = 0.040). Head circumference was not related to the results, although we were able to negatively correlate the birth-weight percentile with the score of the total scale (r = −0.310, *p* = 0.009), emotional scale (r = −0.280, *p* = 0.019), and hyperactivity scale (r = −0.246, *p* = 0.040). Neonatal outcome was not associated with behavioral problems. 

### 3.4. Multivariate Analyses

In order to analyze the mediating factors, we performed a multiple linear regression analysis. Table 6 summarizes the variables included in each model. We included sociodemographic factors in the first model. Maternal employment status (ES) was positively related to global behavioral score (F(1,62) = 5.15, *p* = 0.027). In this regard, unemployed mothers had children with higher scores and, consequently, worse classifications. The R^2^ value was 0.077, showing that 7% of the effect is explained by differences in the mother’s ES. 

In the second model, the birth-weight percentile was negatively related to the total score (F(1,59) = 5.58, *p* = 0.022) in the second model. The R^2^ value was 0.089; therefore, this variable could explain the 8.9% effect.

In the third model, we found that the need for early child support was negatively related to the total score of behavioral problems (F(1,59) = 5.22, *p* = 0.026). These findings could be explained by the higher rates of severe FGR and extreme prematurity among these children, making them more prone to behavioral disabilities. Table 7 sums up these models.

## 4. Discussion

We conducted a study to assess the behavioral development of children with FGR at 6 years of age. We have demonstrated higher behavioral disability rates in these children, specifically, high rates of hyperactivity or conduct problems. At the same time, we have proved a negative relationship between the birth-weight percentile and the total behavioral scale score.

FGR is a cause for concern among obstetricians due to the perinatal morbidities it generates, which are secondary to prematurity or the hemodynamic process itself. In recent decades, the neurodevelopmental deficits of these children, especially behavioral or cognitive impairments, have attracted the attention of both clinicians and researchers.

### 4.1. Regarding the Prevalence of Behavioral Disorders

Our study assessed the possible behavioral problems at the age of 6 (70–84 months) of children previously diagnosed with FGR. At this stage, the children’s attentional capacity has matured to the point where they are able to maintain a state of alertness, their ability to resolve conflicts or problems has increased, and psychosocial relationships with their environment are well established.

Multiple studies have shown that the neurodevelopment of children with FGR during childhood is not comparable to that of children with a normal birth weight. In our study, we found a high prevalence of possible behavioral problems. In fact, 14.3% of the children had abnormal scores on the questionnaire, while 15.7% had borderline scores. Therefore, 30% of the children assessed (abnormal and borderline scores) should undergo more specific evaluations to establish a correct diagnosis. In particular, within the areas studied, the areas of conduct (borderline: 25.7%, abnormal: 11.4%) and hyperactivity (borderline: 8.6%, abnormal: 30%) were the most affected (externalizing problems), while emotional areas and peer problems were less strongly affected (internalizing difficulties). Parents reported that 27.1% of the children had experienced learning difficulties at school.

When evaluating these differences based on the time of FGR diagnosis, early-onset FGR presented worse scores in terms of externalizing problems (behavior and hyperactivity), while late-onset FGR presented worse scores in terms of peer problems and prosocial areas. Similarly, we found a higher percentage of academic difficulties in the subgroup of children diagnosed with early-onset FGR (43.5%) compared to children diagnosed with late-onset FGR (19.6%).

Several studies have shown similar results to ours. Guellec et al. (2011) assessed children at 8 years of age using the SDQ. They identified 33.3% of behavioral problems in FGR infants that were born before 28 weeks of gestational age and 19.1% of behavioral problems in those born between 29 and 32 weeks of gestational age. Similarly, the learning difficulties rates were similar to ours, with 35.5% in those born before 28 weeks of gestational age and 28% in those born between 29 and 32 weeks of gestational age. However, their hyperactivity problem rates varied between 19.1 and 23.5%, while we found 39.1% of hyperactivity disabilities in those children diagnosed before 32 weeks of gestation (data not shown). It is important to note that they classified and evaluated infants by gestational age in their study, without determining which subjects presented real growth restrictions [19]. 

A previous meta-analysis evaluated behavioral and executive function problems in infants born before 33 weeks of gestation or weighing less than 1500 g. Compared to those born at term or at an appropriate weight, the infants displayed worse results in terms of academic achievement, attention, internalizing problems, and executive function. However, the analysis did not differentiate between growth restriction and growth that was appropriate for the subjects’ gestational age [14]. We must note that executive function is a cognitive process and has significant implications for behavioral skills such as self-regulation and inhibition [35].

These findings have also been supported by studies on late-onset FGR children. Geva et al. (2006) showed that FGR children presented a higher incidence of learning disabilities, memory problems, low creativity, and executive function problems compared to children with normal growth [16]. Similarly, Kulseng et al. (2007) found that children born with a very low birth weight at term (less than 1500 gr) presented worse results in terms of attention tasks and executive function at 14 years of age, at around 25% [23]. However, in another study, attention deficit and hyperactivity disorders were slightly higher in underweight children than in the normal-weight population [36]. The authors did not perform a correct discrimination of growth restriction, so it is impossible to compare their findings with those in our study. On the other hand, we did not conduct a specific evaluation of the attentional network. Despite the fact that we found a high rate of attention and hyperactivity problems in our population, a diagnostic test is needed to confirm this finding.

We did not find a relationship between the behavior assessment results and gestational age at delivery, although we did find that children with a lower birth weight had poor results regarding the total, emotional, and hyperactivity scores. This may be due to our sample size. Nevertheless, a previous meta-analysis found that being underweight and premature may lead to attention-deficit hyperactivity disorders [37].

Behavioral disabilities have significant consequences since problems in this area in the preschool years may be associated with a higher prevalence of externalizing and internalizing problems at the age of 10–14 years, leading to learning and adaptation problems [38].

### 4.2. Regarding the Prediction of Doppler Markers and Prematurity

Doppler markers have proven useful in monitoring FGR fetuses to reduce perinatal morbidity and mortality. The umbilical artery (UA) has been the major protagonist in predicting short-term adverse outcomes in this group of children [39,40,41]. However, associations between the changes in UA waves and neurodevelopment, specifically psychosocial and academic development, are controversial [42,43]. In our case, when we evaluated the predictive capacity of the UA in neurodevelopment, we found no association between its percentile and the results on the SDQ, so we could not relate it to behavioral effects. Nonetheless, children with a pathological pulsatility index (PI) for the UA tended to present worse outcomes. These results must be interpreted with caution since we have only a few cases of extreme prematurity and pathological UA waves.

Traditionally, brain sparing has been considered a protective factor to maintain proper brain function in more critical hemodynamic situations. However, this fact is controversial, and multiple studies have shown that it may not entirely be a protective factor. Regarding behavior, Richter et al. (2020) found that the presence of brain sparing was associated with better behavioral outcomes, specifically externalized behavioral profiles (conduct and hyperactivity) [44]. Other studies have failed to link this phenomenon to behavioral issues when assessing children later [45,46]. However, the parents reported more socialization and attention problems in children that had shown brain sparing, although this was not verified with diagnostic tests [45]. In our case, we observed in the bivariate analysis that those children with a pathological CPR had significantly worse scores on the total behavior scale. Emotional, conduct, and hyperactivity areas were also negatively affected by brain sparing, although the values did not reach significance.

The brain-sparing effect has been confirmed by several studies as a hierarchy process, such that when vasodilation is detected in the middle cerebral artery (MCA), this process has already occurred in the anterior cerebral artery (ACA). This hierarchy has been associated with poorer cognitive outcomes, specifically in terms of attention, social interaction, and adaptation abilities [47]. In our case, due to the design of our study, we were not able to verify this hierarchy. However, we observed that children who presented a brain-sparing process (whether by a pathological MCA or CPR) had a higher rate of psychosocial and hyperactivity symptoms.

Despite the importance of redistribution, gestational age at delivery remains the main factor regarding neurological development during childhood [45,48,49]. Nevertheless, the severity of growth restriction may be a risk marker for the appearance of behavioral problems during childhood and adolescence, and, thus, may inform future screening and prevention strategies.

Our study has several strengths. The most important of these is the strict definition of the FGR condition. This allows us not to underestimate our results. Moreover, we have carried out an assessment of children at a late age (6 years of age). At this age, social skills and behavioral problems are well established. Finally, we assessed the children using the SDQ, a validated screening test that allows for the correct discrimination between pathological and non-pathological cases [32,34]. 

Our main limitation was the sample size. As a single-center study, we were unable to locate a large number of children with a history of FGR. Of those cases that were found, a small proportion of parents could not be located or refused to be assessed. Because of our small sample size, the R-square coefficient obtained was low, this being a limitation in our multiple linear regression model. It would explain the low percentage of variability obtained. Furthermore, we could not evaluate a control group without growth restriction to compare the results obtained. Although the behavioral assessment was prospective, the cohort design was retrospective. This fact only provides an association between behavioral outcomes and FGR characteristics. A multicenter study with a larger sample size and a control group is needed in order to verify our findings. 

## 5. Conclusions

We found high behavioral disability rates, particularly in the hyperactivity or conduct areas, in children with FGR. At the same time, we found a negative relationship between the birth-weight percentile and the score of the total behavioral scale. Regarding FGR onset, early-onset FGR children presented poor scores for externalizing problems (behavior and hyperactivity), while late-onset FGR children presented worse scores regarding problems with peers and prosocial areas. No relationship was found between gestational age at delivery and behavioral disabilities. Behavior was not affected by gestational age at delivery, although it was influenced by birth weight. However, the hyperactivity scale was not affected by either birth weight or gestational age at delivery. We observed that children exhibiting brain sparing presented significantly worse results in the total behavior scale, in addition to a negative effect in the emotional, conduct, and hyperactivity areas.

## Figures and Tables

**Table 1:** Inclusion and exclusion criteria.

Inclusion Criteria	Exclusion Criteria
Birth weight less than the 3rd percentile	Structural and chromosomal abnormalities Multiple pregnancies Small for gestational age
Birth weight less than the 10th percentile and abnormal Doppler study: - UA-PI above the 95th percentile - MCA-PI below the 5th percentile - CPR below the 5th percentile, or - Uterine arteries-PI above the 95th percentile	
CPR = MCA-PI/UA-PI	

UA-PI: umbilical artery pulsatility index, MCA-PI: middle cerebral artery pulsatility index, CPR: cerebroplacental ratio, PI: pulsatility index.

**Table 2:** Sociodemographics, delivery characteristics, and neonatal outcome frequencies.

Variables	*n* (%)	Mean ± SD
Separated parents	21 (30)	
Maternal educational level		
Primary school	7 (10)	
Secondary school	38 (54.3)	
Bachelor’s degree	23 (32.9)	
Paternal educational level		
Primary school	18 (25.7)	
Secondary school	36 (51.4)	
Bachelor’s degree	13 (18.6)	
Maternal Unemployed status	25 (35.7)	
Paternal Unemployed status	8 (11.4)	
Socioeconomic status		
Low	10 (14.3)	
Middle	54 (77.1)	
High	4 (5.7)	
Smoker in pregnancy	16 (22.9)	
Postpartum depression	14 (20)	
Pre-eclampsia	24 (34.3)	
Gestational age at diagnosis of FGR (mean)		33.14 ± 4.31
Early onset	23 (32.9)	
Late onset	46 (65.7)	
Fetal weight at diagnosis		1616.38 g ± 660.25
UA PI Percentile (mean)		61.52 ± 27.94
Pathologic	14 (20)	
MCA PI Percentile (mean)		15.69 ± 22.68
Pathologic	29 (41.4)	
CPR percentile (mean)		14.18 ± 22.74
Pathologic	35 (50)	
Vaginal delivery	25 (35.7)	
Cesarean section	45 (64.3)	
Gestational age at delivery		35.61 ± 3.21
<28 weeks	2 (2.9)	
28–32 weeks	10 (14.3)	
32–37 weeks	28 (40)	
>37 weeks	30 (42.9)	
Pathological non-stressant test	31 (44.3)	
Arterial blood cord pH		7.27 ± 0.09
Birthweight (grams)		1848.30 ± 589.74
Gender (female)	37 (52.9)	
Head circumference at delivery (cm)		30.26 ± 3.37
Breastfeeding	52 (74.3)	
Days of NICU admission		127 ± 26.78
Neonatal outcomes		
ARDS	22 (31.4)	
Neonatal sepsis	14 (20)	
ROP	6 (8.6)	
BPD	4 (5.7)	
GMH	3 (4.3)	
PDA	6 (8.6)	
NEC	3 (4.3)	
Intestinal perforation	2 (2.9)	
Acute kidney failure	2 (2.9)	

FGR: fetal growth restriction, UA PI: umbilical artery pulsatility index, MCA PI: middle cerebral artery pulsatility index, CPR: cerebroplacental ratio, SD: standard deviation, NICU: neonatal intensive care unit, ARDS: acute respiratory distress syndrome, ROP: retinopathy of prematurity, BDP: bronchopulmonary dysplasia, GMH: germinal matrix hemorrhage, PDA: patent ductus arteriosus, NEC: necrotizing enterocolitis.

**Table 3:** Percentages of children with behavioral problems.

SDQ Scales	Normal (%)	Borderline (%)	Abnormal (%)
Total difficulties	70	15.7	14.3
Emotional problems	72.9	10	17.1
Conduct problems	62.9	25.7	11.4
Hyperactivity	61.4	8.6	30
Peer problems	78.6	11.4	10
Prosocial	95.7	2.9	1.4

**Table 4:** Percentages of children with behavior problems, depending on FGR onset.

	FGR Onset	Normal	Borderline	Abnormal	
Total	E-O	65.2	17.4	17.4	n/s
	L-O	71.7	15.2	13	
Emotional	E-O	65.2	21.7	13	n/s
	L-O	76.1	4.3	19.6	
Conduct	E-O	60.9	34.8	4.3	n/s
	L-O	63	21.7	15.2	
Hyperactivity	E-O	56.5	4.3	39.1	n/s
	L-O	63	18.9	26.1	
Peer problems	E-O	82.6	13	4.3	n/s
	L-O	76.1	10.9	13	
Prosocial	E-O	100	0	0	n/s
	L-O	93.5	4.3	2.2	

E-O: Early-onset, L-O: Late-onset, n/s: not significant.

**Table 5:** Percentages of children with behavioral problems, depending on Doppler measurements.

	UA	Normal (%)	Borderline (%)	Abnormal (%)	
Total	Pathological	64.3	14.3	21.4	n/s
	Normal	74.5	13.7	11.8	
Emotional	Pathological	64.3	14.3	21.4	n/s
	Normal	74.5	9.8	15.7	
Conduct	Pathological	64.3	28.6	7.1	n/s
	Normal	64.7	23.5	11.8	
Hyperactivity	Pathological	50	14.3	35.7	n/s
	Normal	66.7	7.8	25.5	
Peer problems	Pathological	78.6	14.3	7.1	n/s
	Normal	80.4	7.8	11.8	
Prosocial	Pathological	100	0	0	n/s
	Normal	94.1	3.9	2	
	MCA				
Total	Pathological	69	17.2	13.8	n/s
	Normal	80	13.3	6.7	
Emotional	Pathological	69	13.8	17.2	n/s
	Normal	80	6.7	13.3	
Conduct	Pathological	62.1	31	6.9	n/s
	Normal	70	16.7	13.3	
Hyperactivity	Pathological	65.5	6.9	27.6	n/s
	Normal	66.7	10	23.3	
Peer problems	Pathological	75.9	10.3	13.8	n/s
	Normal	83.3	10	6.7	
Prosocial	Pathological	93.1	6.9	0	n/s
	Normal	96.7	0	3.3	
	CPR				
Total	Pathological	60	22.9	17.1	χ^2^(2, N = 70) = 6.36, *p* = 0.042
	Normal	88.5	11.5	0	
Emotional	Pathological	65.7	14.3	20	n/s
	Normal	88.5	3.8	7.7	
Conduct	Pathological	60	25.7	14.3	n/s
	Normal	69.2	26.9	3.8	
Hyperactivity	Pathological	57.1	11.4	31.4	n/s
	Normal	73.1	3.8	23.1	
Peer problems	Pathological	82.9	8.6	8.6	n/s
	Normal	73.1	15.4	11.5	
Prosocial	Pathological	97.1	0	2.9	n/s
	Normal	92.3	7.7	0	

UA: umbilical artery; MCA: middle cerebral artery; CPR: cerebroplacental ratio; n/s: not significant.

**Table 6:** Variables included in each model.

Model	Variables
1	Separated parents
	Maternal and paternal educational level
	Maternal and paternal employment status
	Socioeconomic status
2	Doppler measurements:
	UA-PI percentile, MCA-PI percentile, and CPR percentile
	Birthweight percentile at delivery
	Gestational age at delivery
3	Gender
	Adverse neonatal outcomes
	Early child support
	Academic difficulties
	Nursery assistance

UA-PI: umbilical artery pulsatility index; MCA-PI: middle cerebral artery pulsatility index; CPR: cerebroplacental ratio.

**Table 7:** Multiple linear regression values of the different models.

Model	Variables	Β	Standard Error	T	95% CI		*p*	R^2^
					Lower	Upper		
1	Maternal employment status	−2.97	1.31	−2.26	−5.60	−0.355	0.027	0.077
	(Constant)	12.95	1.06	12.18	10.82	15.08	<0.001	
2	Birth-weight percentile	−0.659	0.279	−2.36	−1.21	−0.100	0.022	0.089
	(Constant)	11.26	0.697	16.15	9.86	12.66	<0.001	
3	Need for early child support	3.28	1.43	2.28	0.409	6.16	0.026	0.081
	(Constant)	9.6	0.781	12.3	8.04	11.16	<0.001	

Variables with significant value in the different models: maternal employment status (unemployed or active worker), birth-weight percentile, and early child support (yes or no). B: beta standardized coefficient, T: t-value, *p*: *p*-value, R^2^: R-squared value, CI: confidence interval.

## Data Availability

Not applicable.
